# Sympatric speciation in the *Simulium arcticum* s. l. complex (Diptera: Simuliidae): The Rothfels model updated

**DOI:** 10.1002/ece3.5402

**Published:** 2019-07-01

**Authors:** Gerald F. Shields, William S. Procunier

**Affiliations:** ^1^ Department of Life and Environmental Sciences Carroll College Helena Montana; ^2^ Department of Psychology Nipissing University North Bay Ontario Canada

**Keywords:** black flies, geographic distributions, polytene chromosomes, speciation

## Abstract

**Abstract:**

We tested the Rothfels sympatric speciation model for black flies by comparing all available data for sex‐chromosome diversity with the geographic locations of larval collection sites within the *Simulium arcticum* complex of black flies (Diptera: Simuliidae). Five separate data sets equaling about 20,000 larvae were included from throughout the geographic range of this complex. We record a total of 31 taxa having unique sex chromosomes, all of which demonstrate linkage disequilibrium with most taxa sharing autosomal polymorphisms. All siblings share portions of their distributions with *S. negativum*, the presumed oldest member of the complex. Twenty‐one of 22 cytotypes have distributions within the ranges of siblings thus supporting the sympatric speciation model of Rothfels. Chromosomally diverse sites may require analysis of as many as 200 larvae to be properly described. There is no effect of any inversions influencing the occurrence of other inversions. Finally, we report a new cytotype, *Simulium arcticum* IIL‐6, which we originally discovered in Alaska. Aspects of future genomic research are discussed as they relate to the main chromosomal structural/functional tenants of the model.

**OPEN RESEARCH BADGE:**

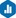

This article has earned an Open Data Badge for making publicly available the digitally‐shareable data necessary to reproduce the reported results. The data are available at https://doi.org/10.6084/m9.figshare.7719398

## 1 INTRODUCTION

The process by which organisms become reproductively isolated, speciation, has been debated since Darwin (1859) published “On the Origin of Species by Natural Selection, or the Preservation of Favoured Races in the Struggle for Life.” Originally, the idea that individuals could become reproductively isolated in the same place (sympatrically) gained favor among biologists because of Darwin's species concept and because of his ideas about natural selection. Gradually, though, support for sympatric speciation waned and in a series of seminal works Mayr (1942, 1963) argued convincingly for a divergence by distance model (allopatry) and against sympatric speciation. Thus, most biologists now envision an allopatric speciation model by which most organisms become reproductively isolated (Coyne & Orr, 2004). This allopatric model has become the conventional way of viewing the mode of speciation. That is, it is true until shown otherwise. Sympatric speciation, however, has not gone away and treatises by White (1973, 1978) principally on Australian morabine grasshoppers and by Bush (1969) and Bush et al., 1989) on the *Rhagoletis* genus of fruit flies have kept the topic relevant. Moreover, there has been a recent resurgence in the speciation debate (Ayala et al., 2011; Feder & Nosil, 2009; Guerrero, Rousset, & Kirkpatrick, 2012; Hoffman & Riesberg, 2008) and Gavrilets (2014) summarizing what we now know about speciation. The works of White and Bush were based on chromosome change in species of insects, yet in their monumental coverage of speciation Coyne and Orr (2004) state that, “It is far from clear whether chromosomal speciation is common in animals generally. Indeed, we know of no compelling evidence for chromosomal speciation (*and by extension*,* sympatric speciation*) in animals.” Doellman et al. (2018) have used molecular approaches in *Rhagoletis* to document host related variation in divergent life history adaptation through increased selection on diapause and reduced gene flow. They suggest host races may be recognized as different genotypic entities and perhaps even good species in sympatry.

Thus, the argument may not be whether sympatric speciation occurs or is even possible but rather if it is widespread. Interpretations of allopatric speciation are clearly both common and widespread in many groups of organisms (Coyne & Orr, 2004; Price, 2008). However, extant allopatric distributions neither confirm nor deny allopatric origins. This is because allopatric distributions make tests of reproductive isolation difficult, if not impossible, by virtue of geographic separation and lack of opportunity to mate. It may be possible for different types of organisms to become reproductively isolated in different ways (Feder et al., 2017)**.** A case in point is the black fly, *Simulium ruficorne* s. l. with its present‐day allopatric distribution of cytotypes/siblings (Cherairia & Adler, 2018 *)*.

Rothfels (1989) proposed a sympatric speciation model for black flies (Simuliidae) based on the somewhat fragmentary data available at that time. Larval black flies possess enlarged polytene chromosomes (Landau, 1962; Rothfels & Nambiar, 1981) and chromosomal rearrangements are relatively easy to describe (Adler, Currie, & Wood, 2004; Rothfels, 1979; Shields & Procunier, 1982). The Rothfels model (1989) was based on: (a) “frequent and often exclusive involvement of changes in the sex‐chromosome systems,” (b) “sex‐chromosome polymorphisms that may display linkage disequilibria,” (c) “complexes that differ only in sex chromosomes and share extensive ancestral autosomal polymorphisms,” (d) “sympatric or widely overlapping distributions of the most closely related species” (taxa), and (e) “species that differ in their biology and perhaps their present‐day distributions.” Of interest is that one of the tenants of the Rothfels model pertaining to chromosomal structural rearrangements and their redistribution is still observed; namely, one and the same inversion/band can be autosomally polymorphic, sex linked, fixed and/or lost within various taxa of the group.

Evolutionary biologists are left with the challenge of analyzing large numbers of genetically identified individuals throughout the current distributional ranges of closely related taxa for clues that might suggest support for either an allopatric or a sympatric origin. In the present context, dispersal distances are important since fidelity to natal sites could allow in situ genetic change. Nothing is known about dispersal distances in members of the *Simulium arcticum* complex but Shields, Christiaens, Luvan, and Hartman (2009) have shown that genotypes of taxa of the *S. arcticum* complex at the Clearwater River in western Montana are essentially identical over a three‐year period. Dispersal distances of less than 15 km seem typical of most species of black flies (Baldwin, West, & Comery, 1975; Bennett, 1963; Moore & Noblet, 1974). Critical in the present context is that female black flies generally do not undergo long‐distance dispersals after emergence (Adler et al., 2004) although it has been shown that river corridor affects chromosome diversity (Shields & Hokit, 2016).

At least 11 species complexes (cytospecies within a single morphospecies) of black flies have been described for North America alone (Adler et al., 2004; Rothfels & Featherston, 1981) and the number increases to 45 complexes world‐wide (Adler, 2019; http://biomia.sites.clemson.edu/pdfs/blackflyinventory.pdf). Moreover, without the original cytogenetic analyses, these complexes of reproductively isolated sibling species might have gone unrecognized (Rothfels, 1979, 1989). The *S. arcticum* Malloch complex of North America is one of the most diverse in existence, second only to the *S. damnosum* complex in Africa (Conflitti, Shields, Murphy, & Currie, 2015b; Shields, 2013). Five sibling species were initially described within the complex (Procunier, 1984; Shields & Procunier, 1982). Four of these (*S. brevicercum*, *S. saxosum*, *S. arcticum* s. s., and *S. negativum*) were later studied in detail and given full species status by Adler et al. (2004), *Simulium arcticum* IIL‐1 has not been studied further. Comparisons of taxa using mitochondrial and nuclear DNAs show that chromosome inversions occur in the initial stages of differentiation when no morphological changes have yet occurred (Conflitti, Shields, Murphy, & Currie, 2015a, 2016). Further, taxa within complexes usually form a continuum from presumably relatively recent cytotypes to full sibling species. Cytotypes are defined as taxa having unique sex‐linked inversions but whose reproductive status is yet undetermined (Adler et al., 2004; Shields, 2013). Nine taxa are given species status and the other 22 have: (a) unique paracentric chromosomal inversions linked to sex, (b) are not monophyletic in phylogenetic trees based on comparative sequences of DNAs (Conflitti, Kratochvil, Spironello, Shields, & Currie, 2010; Conflitti, Shields, & Currie, 2012; Conflitti, Shields, Murphy, & Currie, 2014), and (c) are assumed to be in the early stages of reproductive isolation (Shields, 2013). *S. negativum* is both morphologically unique and monophyletic in our DNA trees (Conflitti, Shields, Murphy, & Currie, 2016). Moreover, it is the oldest extant member of the complex among those analyzed molecularly. Its separation from the remainder of the complex is estimated at 467,500 YBP (Conflitti et al., 2016) and its putative sex‐determining gene or genes is (are) located in the long arm of chromosome I, unlike all other members of the complex (Adler et al., 2004; Shields & Procunier, 1982). Compelling is the original observations of Rothfels (1989) and to a greater extent our own large data set on *Simulium arcticum* that suggest that newly discovered taxa arise almost exclusively within the geographic distributions of other taxa of the complex whose own distributions are large, suggesting a sympatric origin or at minimum arguing against separation by distance before reproductive isolation.

The Rothfels model has not been rigorously tested on a taxon complex throughout its range of distribution. Consequently, we chose to revisit the model using the available data on the *S. arcticum* complex. Herein, we first use the criteria for sympatric speciation originally set forward by Rothfels (1989) on our enlarged data set for the *S. arcticum* complex to revisit the sympatric model. Importantly, we compare sex‐chromosome diversity of all known members of the *S. arcticum* complex from 13 states and six Canadian provinces throughout the distributional range of the complex in North America. The final prediction of the Rothfels model suggested that species should differ in their biology and possibly geographic locations. By using canonical correspondence analysis, we have previously shown that despite significant overlap, all siblings of *S. arcticum* are ecologically unique (Conflitti et al., 2015b). Further, cytotypes are either ecologically unique, are associated with one another, or with particular siblings (Conflitti et al., 2015b). Thus, for this group, ecological and chromosomal differences develop early in lineage formation, suggesting that local adaptation may be involved in diversification (Conflitti et al., 2015b; Pramual, Kuvangkadilok, Jitklang, Tangkawanit, & Adler, 2012; Shields & Kratochvil, 2011).

Secondly, we ask how much analysis is sufficient to accurately describe sex‐chromosome diversity at any collection location. We have analyzed the chromosomes of more than 1,000 individuals from spring collections at four different sites (Shields et al., 2009) and thus were able to complete this analysis here. Thirdly, because nine sibling species and 22 cytotypes have been described on the basis of unique sex‐chromosome morphologies for the *S. arcticum* complex, we questioned whether the break points of one inversion might influence the break points of other inversions. Finally, we report a new cytotype, *S. arcticum* IIL‐6, which we originally discovered in Alaska.

Scientific justification for this research rests on the facts that: (a) a return to the sympatric speciation question originally proposed by Rothfels (1989) seemed warranted, (b) this is the largest data set assembled for a species of black fly in North America, (c) all data are chromosomally verified, and (d) the complex has been sampled throughout its distributional range.

## 2 MATERIALS AND METHODS

### 2.1 Collection of larvae, chromosome staining, and analysis

We used conventional methods of collection, chromosome staining, and analysis (Adler et al., 2004; Currie, 1986; Rothfels & Dunbar, 1953; Shields & Procunier, 1982).

### 2.2 The data sets

Five data sets constitute the known chromosome diversity and geographic distribution of *S. arcticum* in western North America. They include material from: (a) original collections taken in Alaska from 1980 to 1982 (Shields & Procunier, 1982), (b) a portion of the range from which DNA comparisons were made in 2016 (Conflitti et al., 2016), (c) collections made from 2001 to 2018 in western Montana, Idaho, and eastern Washington (Shields et al., 2006; Shields, 2013, 2014, 2015, 2016; Shields & Shields, 2018; and Shields, unpublished), (d) collections made by WSP in western Canada from 1982 to 1983 (Procunier, unpublished), and (d) analyses from the range of distribution of *S. arcticum* (Adler et al., 2004). All five data sets include chromosomally verified larvae. The former three collections have both GPS coordinates and actual numbers of individuals for each taxon of the complex associated with the sites, while the latter two are located only to county of collection site. Actual coordinates for the county data are available from P. H. Adler, Clemson Univ. Original data based on sex‐chromosome type versus geographic locations of collections are archived in Dryad, a publicly accessible repository under the accession number (https://doi.org/10.6084/m9.figshare.7719398).

### 2.3 Changes in sex‐chromosome systems

We simply list taxa whose sex chromosomes differ. In almost all cases, differences in sex chromosomes are absolute (the exception is *S. brevicecum* whose chromosomes are standard (noninverted) in both sexes). For example, in a 3/14/2006 collection of *S. arcticum* from Rock Creek, Missoula County, Montana all females (*n* = 447) had standard, noninverted, chromosomes while all 261 male larvae were either IIL‐9 st/i (*n* = 106) or IIL‐19 st/i (*n* = 155; Shields et al., 2006).

### 2.4 Linkage to sex

For each taxon, we determined whether paracentric chromosomal inversions were linked to either sex. This was possible since for all analyses, the gonads of each larva were placed on the same microscope slide as the polytene chromosomes. A chromosome inversion was judged to be sex‐linked if it was found exclusively, or very nearly so, in one sex or the other. Chromosomal rearrangements were judged to be autosomal if they occurred in each sex equally or nearly so.

### 2.5 Sharing of autosomal polymorphisms

**Figure 1:**
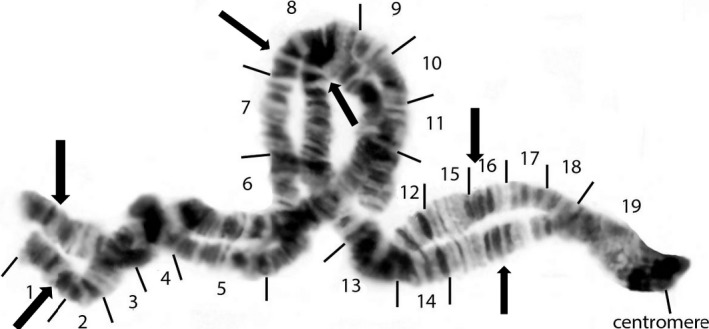
The IS‐1 autosomal inversion in the short arm of chromosome I. Numbers within brackets indicate regions of the entire arm of the chromosome from 1 to 19. Note that the IS‐1 inversion encompasses nearly the central 1/3 of the chromosome and forms a reverse loop (polytene chromosome pairing) from region 6 to region 12. Dark arrows indicate regions of obvious homology

We calculated the proportion of individuals which were heterozygous for the most common autosomal polymorphisms using data sets for which autosomal polymorphisms were chromosomally described or published (Figure 1).

### 2.6 Sex‐chromosome diversity vs. geographic location

For the entire data set, we constructed a database listing the collection site, its geographic coordinates when available, the number of *S. arcticum* taxa present and the number of larvae for each taxon when available. From these data, we could determine the extent of the geographic distributions of all taxa of *S. arcticum*. A key issue was to describe the entire geographic range of each taxon to determine whether there was overlap with other taxa or not. Distributions within geographic ranges might suggest, but do not prove, a sympatric origin. Mutually exclusive distributions might suggest, but do not prove, an allopatric origin.

Locations for 307 sites were determined with varying accuracy. There were 96 sites associated with recent collections using GPS that have an accuracy of plus or minus 5 m. There were 119 legacy sites with good location descriptions that we are confident are within 1 km of the collection location. Finally, 92 sites had poor location descriptions often listing only a county. We used the centroid of the county location for these sites resulting in a spatial accuracy of approximately 100 km. Locations for each chromosome taxon along with associated sibling species and cytotypes were used to create an attribute table.

Following a process similar to the methods used by Swenson and Howard (2005) attribute, data were mapped and analyzed using ArcGIS Pro 2.2. First, point features were created for each sibling species and cytotype. The geographic extent of each species and cytotype was defined using the minimum bounding geometry tool with the convex hull option to minimize the assumed geographic extent of each species/cytotype. It was not possible to use the minimum bounding geometry tool for cytotypes known from fewer than three locations. For these cytotypes, point and line features were created using the buffer tool and a buffer distance of 5 km. This is well within the known dispersal distance of individuals of this species complex (Adler et al., 2004). For all species and cytotypes, the resulting polygons were clipped using a mask of North America. Then, the union tool was used to measure the contact between range extents of each pairwise combination of siblings/cytotypes. Finally, the number of contacts was quantified for each and categorized as a contact with a sibling species or with a cytotype.

### 2.7 How much collection is sufficient?

We had previously analyzed spring larvae from four locations (Blackfoot River, *n* = 774; Little Prickly Pear Creek, *n* = 1,330; Little Blackfoot River, *n* = 1,359 and the Clearwater River, *n* = 2,197) in western Montana to determine reproductive status and continuity of chromosome taxa from year‐to‐year (Shields, 2013; Shields et al., 2009). Given these large sample sizes, we were able to ask the question, how much analysis must be done to fully characterize the black fly cytogenetic diversity at any one site in spring? To assess this statistic, we randomly chose 100 individuals from each collection and determined its taxonomic diversity. We then increased the random sample number to 200, 300, 400, etc. to determine at what sample size the taxonomic diversity failed to increase markedly. We are aware that some taxa of black flies are multivoltine (have more than one generation) so we generally restricted our analyses only to spring, first emergent, larvae.

### 2.8 Do different sex‐linked inversions have at least one breakpoint in common?

More than 80 unique paracentric chromosomal inversions occur within the *S. arcticum* complex, of which 30 are sex‐linked (Shields, 2013; Shields, unpublished). Given one sex‐linked inversion, one wonders whether the probability of a second sex‐linked inversion might be increased if the two inversions have one or the other break points in common. To assess this, we compared all break points of sex‐linked inversions (30 × 30 × 2 = 1,800) to all other break points of other sex‐linked inversions within the complex. If either of the two break points of a sex‐linked inversion was identical to that of another break point of another inversion the taxa compared were given a plus (+), if not, they were given a minus (−).

### 2.9 The IIL‐6 cytotype

Finally, we report the existence of a new cytotype, *S. arcticum* IIL‐6. This cytotype was discovered in the process of analysis of new sites and new drainages in Alaska. It was given cytotypic recognition because the sexes absolutely differed regarding sex chromosomes.

## 3 RESULTS

### 3.1 Changes in sex‐chromosome systems

There is abundant evidence within the *S. arcticum* complex for changes in sex‐chromosome systems (Table 1). With extensive study throughout the range of distribution of *S. arcticum*, there are at least 31 chromosome taxa whose sex‐chromosomes differ. These unique sex chromosomes are the only indication that these taxa differ with the exception of *S. negativum*, which can be morphologically identified (Adler et al., 2004; Shields, 2013).

**Table 1:** Taxa of the *Simulium arcticum* complex. Cytospecies (full sibling species)

*S. arcticum* complex taxon	Sex chromosome constitution	Reference
*S. brevicercum*	Standard	Shields and Procunier (1982) and Adler et al. (2004)
*S. arcticum* IIL‐1	IIL‐1	Shields and Procunier (1982) and Adler et al. (2004)
*S. saxosum*	IIL‐2	Shields and Procunier (1982) and Adler et al. (2004)
*S. arcticum* sensu stricto	IIL‐3	Shields and Procunier (1982) and Adler et al. (2004)
*S. apricarium*	IIL‐7	Adler et al. (2004)
*S. vampirum*	IIL‐8, IIS‐10·11	Procunier (1984) and Adler et al. (2004)
*S. chromatinum*	IIL‐11	Adler et al. (2004)
*S. arcticum* IIS‐4	IIS‐4	Procunier (1984) and Adler et al. (2004)
*S. negativum*	IL‐3·4	Shields and Procunier (1982) and Adler et al. (2004)

Cytotypes			
Number	Sex‐chromosome constitution	Location	Reference
1	*S. arcticum* IIS‐49‐52	Northern Nevada	Adler et al. (2004)
2	*S. arcticum* IIL‐57‐58	Metolius River, Oregon	Adler et al. (2004)
3	*S. arcticum* IIL13	Rock Creek, Montana	Adler et al. (2004) and Shields (2013)
4	*S. arcticum* IIL‐9	Northwestern Montana	Adler et al. (2004) and Shields (2013)
5	*S. arcticum* IIL‐10	Central Montana, Fergus Co.	Adler et al. (2004) and Shields (2013)
6	*S. arcticum* IIL‐14	Great Bear Lake, NW Territories	Adler et al. (2004)
7	*S. arcticum* IIL‐15	South Central Montana	Adler et al. (2004); Shields (2013)
8	*S. arcticum* IIL‐16	Northwest Territories	Adler et al. (2004)
9	*S. arcticum* IIL‐17	Rock Creek, Montana Selway River, Idaho	Adler et al. (2004) and Shields (2013) Shields and Shields (2018)
10	*S. arcticum* IIL‐12	California, Oregon, Northwest Territories	Adler et al. (2004)
11	*S. arcticum* IIL‐6	Delta Clearwater River, Monument Creek, Alaska	This publication
12	*S. arcticum* IIL‐18	West Central Montana	Shields (2013)
13	*S. arcticum* IIS‐12	Boulder River, Sweet Grass County, Montana	Shields (2013)
14	*S. arcticum* IIL‐21	Latah Creek, Spokane Co., Washington	Shields and Kratochvil (2011)
15	*S. arcticum* IIL‐22	Clearwater River, Missoula Co., Montana	Shields (2013)
16	*S. arcticum* IIL‐68	Trout Creek, Lewis and Clark Co., Montana	Conflitti et al. (2010) and Shields (2013)
17	*S. arcticum* IIL‐19	Blackfoot, Clearwater rivers Rock Creek, Montana	Shields et al. (2006)
18	*S. arcticum* IIL‐73·74	Wise River, Beaverhead Co., Montana	Conflitti et al. (2010) and Shields (2013)
19	*S. arcticum* IIL‐79	Central Idaho	Shields (2014)
20	*S. arcticum* IIS‐15	Kootenai River, Lincoln Co., Montana	Shields (2014)
21	*S. arcticum* IIL‐ dup.	Boulder River, Sweet Grass Co., Montana	Shields (unpublished)
22	*S. arcticum* IIL‐80	Asotin Cr., Tucannon River, Columbia Co., Washington	Shields and Shields (2018)

### 3.2 Inversions linked to sex

The majority of taxa within the *S. arcticum* complex display complete linkage to sex and thus linkage disequilibrium (Table 2). In fact, of the seven taxa that do not display complete linkage to sex, none has a linkage to the Y chromosome of less than 0.967 (Table 2).

**Table 2:** Unique paracentric chromosomal inversions and their linkage to maleness in the *S. arcticum* complex^a^

Taxon	Sites observed	Male larvae analyzed	Extent of Y linkage
*Simulium arcticum* IIL‐1	5	278	1.000
*S. arcticum* s. s. IIL‐3	36	2,753	0.998
*S. arcticum* IIL‐6	2	113	1.000
*S. arcticum* IIL‐9	17	479	0.981
*S. arcticum* IIL‐10	9	296	1.000
S. arcticum IIL‐13	4	25	1.000
*S. arcticum* IIL‐15	12	80	1.000
*S. arcticum* IIL‐17	2	82	0.975
*S. arcticum* IIL‐18	12	183	0.967
*S. arcticum* IIL‐19	5	500	0.994
*S. arcticum* IIL‐21	1	86	1.000
*S. arcticum* IIL‐22	3	261	0.996
*S. arcticum* IIL‐38	1	27	1.000
*S. arcticum* IIL‐51	1	16	1.000
*S. arcticum* IIL‐68	3	106	1.000
*S. arcticum* IIL‐ 73.74	2	159	1.000
*S. arcticum* IIL‐79	8	221	0.996
*S. arcticum* IIL‐80	3	21	1.000
*S. arcticum* IIL‐duplication	1	21	1.000
*S. arcticum* IIS‐12	1	17	1.000
*S. arcticum* IIS‐15	1	24	1.000
*S. negativum* IL‐3·4	7	216	1.000
*S. arcticum* IIS‐49‐52	1	n.a.	1.000
*S.arcticum* IIL‐57‐58	1	n.a.	1.000
*S. arcticum* IIL‐14	1	n.a.	1.000
*S. arcticum* IIL‐16	1	n.a.	1.000
*S. arcticum* IIL‐12	1	n.a.	1.000

a *S. brevicercum* is IIL standard,* S. saxosum* (IIL‐2) and *S. apricarium* (IIL‐7) do not have strict Y chromosome sex‐determination. n.a. indicates numbers not available. Data are from Shields and Procunier (1982), Shields (2013), Shields (unpublished), and Adler et al. (2004).

### 3.3 Sharing of autosomal polymorphisms

It is abundantly clear that taxa within the *S. arcticum* complex share autosomal polymorphisms (Table 3). Here, we report the proportions of heterozygotes among some *S. arcticum* taxa. The autosomal polymorphism, IS‐1, occurs in 13 of 15 taxa, while the autosomal inversion, IL‐1, occurs in 11 of 15 taxa.

**Table 3:** Proportions of some shared autosomal polymorphisms among some members of the *Simulium arcticum* complex

Taxon	IS‐1 Inversion	IL‐1 Inversion	IIL‐20 Inversion
*S. brevicercum*	0.039	0.055	0.190
*S. saxosum*	0.022	0.174	0.060
*S. arcticum* s. s.	0.497	0.028	0.595
*S. apricarium*	0.004	0.147	0
*S. negativum*	0.079	0	0
*S. arcticum* IIL‐9	0.088	0.055	0.071
*S. arcticum* IIL‐13	0.004	0.028	0
*S. arcticum* IIL‐15	0	0	0.012
*S. arcticum* IIL‐18	0.002	0	0
*S. arcticum* IIL‐19	0.041	0.294	0
*S. arcticum* IIL‐21	0	0.174	0
*S. arcticum* IIL‐22	0.142	0	0
*S. arcticum* IIL‐73·74	0.015	0.046	0
*S. arcticum* IIL‐79	0.041	0.009	0.060
*S. arcticum* IIL‐6	0.457	0.005	0

### 3.4 Sex‐chromosome diversity vs. geographic distribution

Current distributions of siblings within the *S. arcticum* complex overlap extensively (Table 4). In the majority of cases, the distributions of all siblings overlap with the distributions of other siblings. Moreover, distributions of cytotypes, save one, are all within the distributions of siblings (Table 4).

**Table 4:** Geographic areas and overlap of siblings and cytotypes within the *S. arcticum* complex

Sibling	Distribution Sq. km	No. contacts w/siblings	No. contacts w/cytotypes
*S. saxosum* IIL‐2	2,829,500	8	16
*S. negativum* *IL‐3·4*	2,819,090	8	18
*S. arcticum* *IIL‐1*	2,770,444	7	7
*S. brevicercum* *(IIL‐st/st)*	2,312,022	8	18
*S. apricarium* *IIL‐7*	1,950,914	6	15
*S. arcticum s.s. IIL‐3*	1,683,894	8	14
*S. chromatinum* *IIL‐11*	812,216	5	3
*S. vampirum* *IIL‐8*,* IIS‐10·11*	605,393	6	0
*S. arcticum* *IIS‐4*	15,736	6	0
Cytotypes			
IIL‐9	80,355	7	11
IIL‐13	39,902	6	5
IIL‐15	30,369	5	6
IIL‐10	25,729	5	4
IIL‐79	20,384	6	3
IIL‐19	14,432	6	5
IIL‐18	8,193	5	5
IIL‐22	2,776	6	3
IIL‐6	1,197	3	0
IIL‐80	1,095	6	2
IIL‐12	729	1	0
IIS‐15	441	5	3
IIL‐73.74	81	4	2
IIL‐17	79	5	2
IIL‐21	79	4	1
IIS‐12	79	3	1
IIL‐dup.	79	3	1
IIS‐49·52	79	3	0
IIL‐57·58	79	3	0
IIL‐14	79	0	0
IIL‐16	79	1	0
IIL‐68	8	4	2

### 3.5 What is the appropriate sample size?

**Figure 2:**
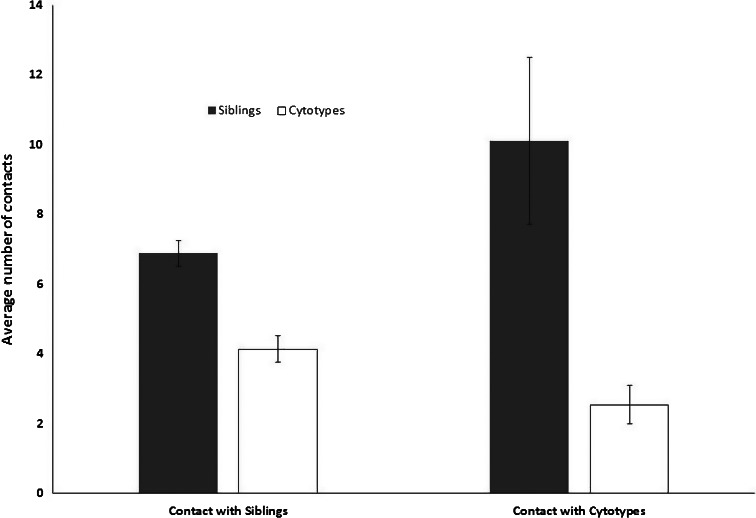
The statistic indicated is the standard error. The average number of chromosome taxa with overlapping (contact) ranges is grouped as: (1) siblings with other siblings, (2) siblings with cytotypes, (3) cytotypes with siblings, and (4) cytotypes with other cytotypes. Note that because siblings have much larger ranges than cytotypes, the average number of contacts that siblings have with cytotype is larger than the number of contacts cytotypes have with siblings

**Figure 3:**
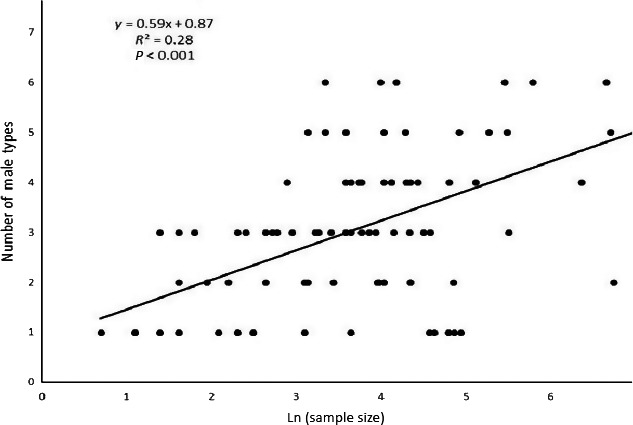
The number of different Y chromosomes plotted against the log of the sample size

**Figure 4:**
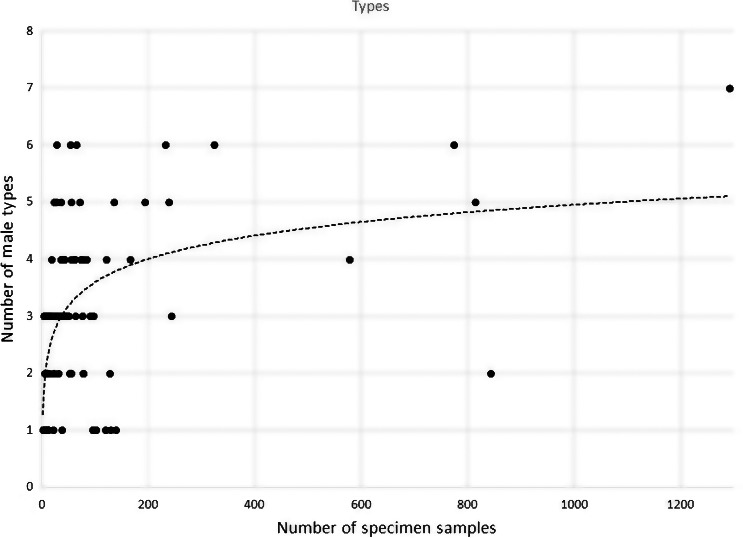
Diversity of taxon types at any collection site versus the sample size of the collections. After 200 samples have been analyzed the diversity increases only slightly.

For spring collections, our data suggest that detected diversity of sex chromosomes reaches about 90% after about 200 samples are analyzed and that detected diversity increases very slowly after that (Figures 2, 3, 4). It is important to acknowledge that our comparisons are all from spring collections and presumably from first generation larvae.

### 3.6 Chromosomal break points

Only 46 of 1800 (2.6%) possible break points were in common (Table 5A–C). Moreover, of the 46 break points in common with others, there appears to be no obvious break point “hot spot.” This may imply that the breakpoint of any inversion has little to no effect on subsequent break points of inversions in the complex**.**

**Table 5:** (A) Distal–distal (away from the centromere) break points in common of sex‐chromosome inversions within the *Simulium arcticum* complex. (B) Proximal–proximal (closer to centromere) break points of sex chromosomes in common within the *S. arcticum* complex. (C) Proximal–distal, distal–proximal break points of sex chromosomes in common within the *S. arcticum* complex

(A)											
	IIL‐1	IIL‐15	IIL‐3	IIL‐22	IIL‐6	IIL‐10	IIL‐68	IIL‐13	IIL‐16	IIL‐17	IIL‐19
IIL‐1		+	−	−	−	−	−	−	−	−	−
IIL‐15	+		−	−	−	−	−	−	−	−	−
IIL‐3	−	−		+	+	−	−	−	−	−	−
IIL‐22	−	−	+		+	−	−	−	−	−	−
IIL‐6	−	−	+	+		−	−	−	−	−	−
IIL‐10	−	−	−	−	−		+	−	−	−	−
IIL‐68	−	−	−	−	−	+		−	−	−	−
IIL‐13	−	−	−	−	−	−	−		+	+	+
IIL‐16	−	−	−	−	−	−	−	+		+	+
IIL‐17	−	−	−	−	−	−	−	+	+		+
IIL‐19	−	−	−	−	−	−	−	+	+	+	

(B)									
	IIL‐1	IIL‐68	IIL‐3	IIL‐80	IIL‐6	IIL‐7	IIL‐13	IIL‐79	IIL‐8
IIL‐1		**+**	−	−	**+**	−	−	−	−
IIL‐68	**+**		−	−	**+**	−	−	−	−
IIL‐3	−	−		**+**	−	−	−	−	−
IIL‐80	−	−	**+**		−	−	−	−	**+**
IIL‐6	**+**	**+**	−	−		−	−	−	−
IIL‐7	−	−	−	−	−		**+**	**+**	−
IIL‐13	−	−	−	−	−	**+**		**+**	−
IIL‐79	−	−	−	−	−	**+**	**+**		−
IIL‐8	−	−	−	**+**	−	−	−	−	
	**IIL‐9**	**IIL‐22**	**IIL‐10**	**IIL‐18**	**IIL‐11**	**IIL‐15**	**IIL‐14**	**IIL‐73**	**IIL‐57·58**
IIL‐9		**+**	−	−	−	−	−	−	−
IIL‐22	**+**		−	−	−	−	−	−	−
IIL‐10	−	−		**+**	−	−	−	−	−
IIL‐18	−	−	**+**		−	−	−	−	−
IIL‐11	−	−	−	−		**+**	−	−	−
IIL‐15	−	−	−	−	**+**		−	−	**+**
IIL‐14	−	−	−	−	−	−		**+**	−
IIL‐73	−	−	−	−	−	−	**+**		−
IIL‐57·58	−	−	−	−	−	**+**	−	−	
		**IIL‐57·58**		**IIL‐21**		**IIL‐12**		**IIL‐74**	
IIL‐57·58				−		−		**+**	
IIL‐21		−				**+**		−	
IIL‐12		−		**+**				−	
IIL‐74		**+**		−		−			

(C)									
	IIL‐3	IIL‐15	IIL‐73	IIL‐6	IIL‐11	IIL‐21	IIL‐7	IIL‐12	IIL‐13
IIL‐3		**+**	**+**	−	−	−	−	−	−
IIL‐15	**+**		−	**+**	−	−	−	−	−
IIL‐73	**+**	−		−	−	−	−	−	−
IIL‐6	−	**+**	−		**+**	**+**	−	−	−
IIL‐11	−	−	−	**+**		−	−	−	−
IIL‐21	−	−	−	**+**	−		−	−	−
IIL‐7	−	−	−	−	−	−		**+**	−
IIL‐12	−	−	−	−	−	−	**+**		**+**
IIL‐13	−	−	−	−	−	−	−	**+**	
	**IIL‐15**	**IIL‐80**	**IIL‐16**	**IIL‐68**	**IIL‐18**	**IIL‐57·58**	**IIL‐1**	**IIL‐22**	**IIL‐74**
IIL‐15		**+**	−	−	−	−	−	−	−
IIL‐80	**+**		−	−	**+**	−	−	−	−
IIL‐16	−	−		**+**	−	−	−	−	−
IIL‐68	−	−	**+**		−	−	−	−	−
IIL‐18	−	**+**	−	−		**+**	−	−	−
IIL‐57·58	−	−	−	−	**+**		−	−	−
IIL‐1	−	−	−	−	−	−		−	−
IIL‐21	−	−	−	**+**	−	−	**+**		−
IIL‐22	**+**	**+**	−	−	−	−	−	−	
IIL‐74	−	**+**	−	−	**+**	−	−	−	−

### 3.7 The IIL‐6 cytotype

**Figure 5:**
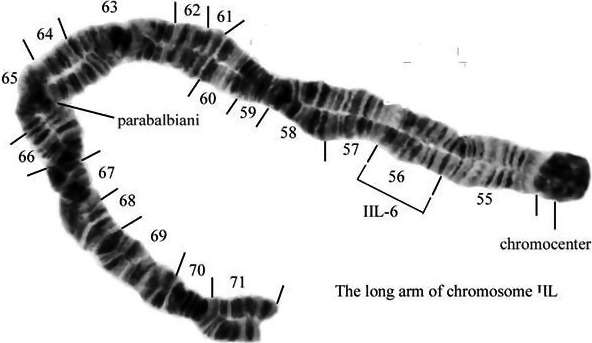
The long arm of chromosome II of the *Simulium arcticum* complex. Lines (55–71) indicate specific sections of the chromosome. The bracket indicates the limits of the IIL‐6 inversion

Of 206 larvae analyzed from the Delta Clearwater River and Monument Creek sites in Alaska, all males (*n* = 113) were heterozygous for the IIL‐6 paracentric inversion while all females (*n* = 93) possessed the standard (noninverted) sequence (Table 6 and Figure 5). This sex‐linked chromosomal sequence inverts the entire section 56 of the long arm of chromosome II (Figure 5). Table 7 lists the most common autosomal polymorphisms in populations of IIL‐6.

**Table 6:** Sex chromosomes of the IIL‐6 *S. arcticum* cytotype

Location	Latitude	Longitude	Elevation (m)	Females X_0_ X_0_	Males *X*_0_ *Y*_IIL‐6_	Polyploid^a^ X_0_ X_0_ Y_IIL‐6_
Delta Clearwater River, Alaska	63.959281	−145.647495	411.8	83	102	1
Monument Creek, Alaska	65.051123	−146.030510	388.0	10	10	0

a One of the three constituents of the triploid was inverted for the IIL‐6 sequence, the other two chromosomes were standard for the IIL‐6 sequence. The presence of sperm in the male gonad indicated that the Y chromosome was male determining as in other black flies (Rothfels & Nambiar, 1981) and unlike *Drosophila*.

**Table 7:** Proportions of heterozygotes for autosomal polymorphisms of the *S. arcticum* IIL‐6 cytotype

Cytotype	1S‐1	IL‐1	IL‐2	IIS‐3	IIS‐4
IIL‐6 (*n* = 206)	0.457	0.005	0.005	0.005	0.047

Note

*S. arcticum* IIL‐6 is also characterized by meiotic development in mature male larvae to sperm and the absence of B chromosomes.

## 4 DISCUSSION

### 4.1 Changes in sex‐chromosome systems

Rothfels (1989) predicted that morphospecies, when investigated cytologically, would be composed of groups that differ in their sex‐chromosome systems. Those predications were based, in part, on early work on the *Simulium arcticum* complex that at that time was known to be composed of five sibling species based on analysis of only 1,254 individuals from only 18 sites in Alaska (Shields & Procunier, 1982). By increasing our sample size about 16‐fold to include individuals from throughout the range of the complex, we have increased the number of taxa to 31. Thus, the first criterion of the Rothfels model has been fulfilled. This criterion appears to be an important characteristic of the *S. arcticum* system and to many complexes of black flies (Adler et al., 2004). Not only are numerous changes (based on paracentric chromosomal inversions) obvious but also many of these changes appear to be absolute (only in one sex or the other). Our research indicates that there are nine siblings and 22 cytotypes within the complex. More chromosome taxa may be discovered in areas not extensively studied here (Yukon Territories, western slopes of the Rocky Mountains in Oregon, Utah, and Colorado, portions of Arizona and New Mexico and possibly in Mexico).

### 4.2 Linkage disequilibrium

The Rothfels model also predicted that changes in sex‐chromosome systems would result in linkage disequilibrium between the sex chromosomes. Linkage to sex, particularly in males, is almost absolute in taxa of the *S. arcticum* complex. In the majority of types, linkage to sex is 100% and in those that are not, linkage is only slightly less than 100%. Thus, the second criterion of the Rothfels model is fulfilled. This is a characteristic common in many other species complexes of black flies (Adler et al., 2004).

### 4.3 Autosomal polymorphisms

The sharing of autosomal polymorphisms between taxa (the third criterion of Rothfels) of the *S. arcticum* complex is obvious. In fact, two of the autosomal polymorphisms (IS‐1 and IL‐1) are shared by a majority of taxa analyzed (13 of 15 in the case of 1S‐1 and 11 of 15 in the case of IL‐1). Moreover, *S. brevicercum*, *S. saxosum*, *S. arcticum* s. s., *S. arcticum* IIL‐9 and *S. arcticum* IIL‐79 have all three autosomal polymorphisms in common. Rothfels (1979) stated that “one taxon's sex‐linked inversion may be another taxon's autosomal inversion.” Indeed, there is evidence for this phenomenon in our data set. In our collection of 5/18/2003 at the Blackfoot River that was chromosomally and morphologically identified as *S. negativum*, 72 of 88 males were also heterozygous for the IS‐1 inversion (Shields et al., 2006). Thus, there may be two types of sex chromosomes at the Blackfoot River in mid‐May.

We have suggested via molecular analysis that gene flow occurs between siblings of black flies (Conflitti et al., 2016). One criterion of the Rothfels model was sibling retention of autosomal polymorphisms. Gene flow and retention of autosomal polymorphisms may be indicative of one‐and‐the‐same process. Possibly, what the molecular analyses indicate is related to what is seen in cytogenetic analyses, that gene flow and retention of autosomal polymorphisms may result in similar observations. However, it may be difficult to separate gene flow from retention of autosomal polymorphisms in closely related types. Whether taxa are designated good species or not seems irrelevant in the present context. What is relevant is that chromosomal inversions linked to sex in males occur before complete reproductive isolation. Indeed, it is the first criterion that differentiates types. We do not discover new types outside the geographic distributions of previously described types. If that were true, we would favor an allopatric model of speciation in these black flies.

### 4.4 Diversity vs. geographic structure

There is wide overlapping of the distributions of *S. arcticum* taxa. The present‐day distributions of all siblings of the complex overlap with other siblings. Indeed, the distribution of *S. negativum*, the presumed oldest member of the complex, overlaps with all other siblings. Also, the distribution of *S. negativum* overlaps with the largest number of cytotypes, further bolstering Rothfels' model. Notable is the fact that the distributions of all cytotypes occur within the ranges of siblings. This fulfills the fourth criterion of the Rothfels model, that there would be frequent sympatric and widely overlapping distributions of the most closely related taxa.

### 4.5 How much analysis is necessary?

Sex‐chromosome diversity at sites extensively analyzed rose dramatically up to a sample size of 200. After that, diversity rose slowly and only about 10% diversity was added if 600 larvae were analyzed. This suggests that most black fly sites (even in this study) are insufficiently analyzed.

Chromosomally diverse sites will require more analysis than taxon‐pure sites. For example, the Little Blackfoot River at Elliston, Montana is one of the most diverse sites we know of with two sibling species and nine cytotypes present. In order to detect all of this diversity, we had to analyze nearly 800 individuals on 4 April 2011 (Shields, 2013). Alternatively, the Cle Elum River site in Washington state is one of only several taxon‐pure sites with only *S. saxosum* being present there. By analyzing 220 individuals from a 24 March 2008 collection, there we found the same diversity we had found with three previous spring collections (*n* = 75, Shields & Kratochvil, 2011).

### 4.6 Break points

The basal region of the long arm chromosome II may have “hot spots” for sex‐linked inversions. Given this, we questioned why this region had so many sex‐linked inversions. By extension, does a one inversion breakpoint increase the probability of another breakpoint? Only 46 of a possible 1800 (2.55%) break points are in common. This suggests that one breakpoint may not influence another breakpoint. The 46 break points in common among taxa may be an overestimate because it is difficult to determine specifically the exact breakpoint in regions between obvious chromosome bands. Some regions of the long arm of chromosome II are so‐called “puffing regions” and since there are few specific and detailed markers in these regions, our analyses might be inaccurate. If break points appeared similar in these regions they were scored as positives. Molecular studies of the basal portion of the IIL region might reveal why so many inversions are located there (Adler et al., 2016). Such an approach should reveal potential epigenomic/genomic interactions as they may apply to gene regulation where topologically associating domains (TAD's; Spielmann, Lupiáñez, & Mundlos, 2018) have been shown to play a functional role in gene expression/regulation and where rearrangement break points across vertebrate species are strongly enriched at TAD boundaries and depleted within TAD's across species (Krefting, Andrade‐Navarro, & Ibn‐Salem, 2018).

### 4.7 The IIL‐6 cytotype

This cytotype was found at only two sites, the Delta Clearwater River and Monument Creek of the Fairbanks‐North Star Borough, Alaska (Table 5). These sites are only 97.37 km (60.5 miles) apart. It is possible for gravid females of at least some species of black flies disperse this distance (Adler et al., 2004). Figure 5 maps the IIL‐6 inversion on the long arm of chromosome II. Three collections at the near‐by Chena and Salcha rivers were exclusively *S. negativum* and not *S. arcticum* IIL‐6 (Shields & Procunier, 1982).

### 4.8 Caveats about the data

Although we claim to have analyzed *S. arcticum* larvae throughout the general range of distribution, we have not analyzed larvae from every drainage. It is likely that *S. arcticum* can be found in additional unstudied drainages. The large majority of samples (72%) are precisely located. For those that are not, we used the centroid of the county resulting in a special accuracy of from 10 to 100 km. This does not affect the outcome of the analysis since the overall distribution of the species complex covers tens of thousands of square miles.

Second, the current distributions of taxa within the *S. arcticum* complex may give little, if any indication, of the process or processes by which these taxa have become reproductively isolated. It is likely that taxa have undergone additional evolutionary processes since they originally arose. Rothfels (1989) argues that continentally distributed taxa may be difficult to interpret as to type of origin since sympatry was the reason they were detected in the first place. However, the number of taxa within the *S. arcticum* complex decreases to the north, east, and south in a fashion similar to that of *Prosimulium (Helodon) onychodactylum* (Henderson, 1986a, 1986b; Newman, 1983) and *S. tuberosum* in eastern North America (Landau, 1962; Mason, 1982, 1984) suggesting that the Rocky Mountain region may have been influenced by past glacial activity providing refugia for new types to exploit.

Most cytotypes occur in the mountainous regions of the study area. Presumably, new cytotypes have expanded their ranges to new habitats created by ice‐age decline. We assume that both choice of egg‐laying sites by adult females and larval segregation in various microhabitats are occurring. The Rothfels model can be tested further by additional detailed study of other complexes of black flies.

There is no evidence that any taxa of *S. arcticum* complex occur in the Old World nor is there any record of the *S. arcticum* complex in Mexico (Adler et al., 2004). *S. apricarium*, a sibling of the *S. arcticum* complex, occurs in California, Arizona, and New Mexico (Adler et al., 2004) and *S. chromatinum*, also a sibling of the *S. arcticum* complex, occurs in Arizona and New Mexico (Adler et al., 2004). *S. vandalicum* of the *S. tuberosum* complex and *S. carbunculum* of the *S. pugetense* complex, both relatives of *S. arcticum*, occur in Mexico (Adler et al., 2004). Additional collecting there may reveal the presence of *S. arcticum*.

Our data sets are somewhat uneven in that the northwestern regions of the United States and Canada and possibly Alaska have been reasonably well researched, whereas portions of the Yukon and southern range of distribution of *S. arcticum* have only been lightly sampled. In this southwestern region it is entirely possible that heretofore unstudied taxa of the *S. arcticum* complex may occur.

The original morphospecies of classical black fly taxonomy almost always becomes a complex of sibling species when detailed cytogenetic analyses are conducted. As suggested in this study, types might arise from preexisting types through chromosome rearrangements in sympatry. This may be a necessary condition for sympatric speciation.

## 5 SUMMARY

All of the criteria set forward by Rothfels in his sympatric speciation model have been fulfilled by additional sampling and analysis. What may never be known is whether present‐day distributions of taxa within the complex reflect origins. However, the weight of evidence suggests that taxa of the *S. arcticum* complex may give rise to new taxa via a sympatric model. Our observations also suggest that extensive sampling and analysis are necessary to adequately characterize the chromosome diversity at any one site and that break points of inversions seem not to influence the origin of additional inversions. Future genomic 3D analysis (Adler, Hamada, Nascimento, & Grillet, 2017) of inversion hotspots within the complex should prove invaluable for defining the potential structural integrity/molecular makeup of inversions and their potential functions.

One implication of these results is that chromosomal change (including inversions) and the consequent lack of recombination within inverted regions may give rise to sex‐chromosome complexes that subsequently diverge. We emphasize that our results relate only to black flies of the *S. arcticum* complex. A similar study may be difficult in organisms other than black flies since many of those organisms do not possess polytene chromosomes and the detail seen here may not be possible.

## CONFLICT OF INTEREST

None declared.

## AUTHORS CONTRIBUTIONS

GFS conceived the idea, collected most of the material, assembled and analyzed the data, wrote the original manuscript and responded to reviewers. WSP collected and analyzed some of the data and contributed to revising the manuscript.

## Data Availability

Original data based on sex‐chromosome type versus geographic locations of collections are archived in Dryad, a publicly accessible repository under the accession number (https://doi.org/10.6084/m9.figshare.7719398).
